# Anti-inflammatory activity of benidipine hydrochloride in LPS-activated mammalian macrophages

**DOI:** 10.1007/s00210-024-02989-w

**Published:** 2024-02-05

**Authors:** Hülya Servi, Tanya Beril Korkmaz, Furkan Ayaz

**Affiliations:** 1 Faculty of Science, Department of Biotechnology, Mersin University, Mersin, Turkey; 2 Department of Molecular Biology and Genetics, Faculty of Engineering and Natural Sciences, Biruni University, Istanbul, 34010 Turkey

**Keywords:** Hypertension, Calcium channel blockers, Benidipine hydrochloride, Macrophage, Inflammation

## Abstract

Benidipine hydrochloride (BH), a medication frequently used by the hypertension patients, acts as a calcium channel blocker. However, its effects on the macrophages have not been investigated thus far. Our goal was investigating the effect of the benidipine hydrochloride to modulate the J774.2 murine macrophage cells inflammatory activity. Our results suggest that in the absence of a standard stimulating agent (LPS) BH did not stimulate the macrophages to produce pro-inflammatory IL-12p40, TNF-α, GM-CSF and IL-6 cytokines. However, when BH was administrated to the cells in the presence of LPS as stimulating agent, it reduced the production of these pro-inflammatory cytokines. Therefore, it had anti-inflammatory activity. At the clinical setting this study suggests that BH can be utilized as hypertension drug that can suppress the inflammation associated with it.

## Introductıon

Hypertension (HT) is a medical condition in which patients experience elevated blood pressure within the arteries. Hypertension typically involves a combination of acquired, genetic, and metabolic factors (Gabb 2020). Blood pressure in patients with HT is regulated by pharmacological treatment. (Carey et al. 2022; Kitt et al. 2019).

Antihypertensive medications reduce blood pressure by dilating blood vessels, preventing constriction, or decreasing the workload on the heart muscle (Esam et al. 2021). These medications can be generally categorized into seven groups: ACE (angiotensin-converting enzyme) inhibitors, diuretics, angiotensin receptor blockers, alpha-blockers, calcium channel blockers, beta-blockers, and centrally acting drugs (Kitt et al. 2019).

Calcium channel blockers act as vasodilators by obstructing the calcium ions responsible for vessel contraction or constriction (Akizuki et al. 2008; Yao et al. 2006; Esam et al. 2021). Benidipine hydrochloride (BH) belongs to the class of dihydropyridine calcium channel blockers and has been widely used to treat hypertension (McDonough et al. 2020). The primary pharmacological mechanism underlying BH's antihypertensive effect is its ability to relax vascular smooth muscle (VSM) and myocardium cells by blocking L-type voltage-dependent calcium channels (LVDCCs). BH has the ability to block not only L-type voltage-dependent calcium channels (LVDCCs) but also other ion channels that facilitate the entry of calcium ions (Ca2+) (Yang et al. 2019). Both toxicity studies and clinical reports have consistently demonstrated that BH is biocompatible across a broad range of dosage levels (Martinez et al. 2023; Sofy et al. 2020).

In this study, the immunostimulatory and immunomodulatory activities of BH were investigated on mammalian macrophages in the absence and presence of LPS as a stimulant, respectively. In a clinical setting, it will be important to have knowledge about BH’s possible pro or anti-inflammatory activities for its most efficient utilization. If it suppresses inflammation, patients who have acute inflammation associated with hypertension should not be prescribed additional anti-inflammatory drugs to prevent unwanted side effects (Patrick et al. 2021; De Miguel et al. 2015; Xiao and Harrison 2020).

Due to the growing number of studies associating inflammation and hypertension, we aimed to investigate the immunomodulatory and immunotoxic activities of BH on mammalian macrophages (Patrick et al. 2021; De Miguel et al. 2015; Xiao and Harrison 2020). In a clinical setting, it is crucial to understand BH’s potential pro or anti-inflammatory activities for its optimal utilization. If BH exhibits inflammatory properties, caution should be exercised regarding its use in patients suffering from hypertension associated with inflammation (Patrick et al. 2021; De Miguel et al. 2015; Xiao and Harrison 2020). Conversely, if BH suppresses inflammation, patients with acute inflammation related to hypertension should not be prescribed additional anti-inflammatory drugs to prevent unwanted side effects (Patrick et al. 2021; De Miguel et al. 2015; Xiao and Harrison 2020).

In this study, we investigated the immunostimulatory and immunomodulatory activities of BH on mammalian macrophages in the absence and presence of LPS as a stimulant, respectively. Our results suggest that BH exhibits anti-inflammatory activity on stimulated macrophages. In a clinical setting, inflammation-associated hypertension can potentially be treated without the administration of anti-inflammatory drugs if BH is prescribed to the patients (Patrick et al. 2021; De Miguel et al. 2015; Xiao and Harrison 2020).

## Materıals and methods

### J774.2 macrophage cell culture

The J774.2 macrophage cell line was originally acquired from ATCC. Roswell Park Memorial Institute (RPMI) medium was used for cell propagation. Additionally, the culture medium contained 10% fetal bovine serum (FBS) and 1% antibiotic solution (100 μg/mL penicillin-streptomycin) to support efficient growth without contamination. The cells were incubated in a humidified 37°C CO2 incubator with 5% CO2. Established protocols from our previous studies were utilized for cell culture (Xiao and Harrison 2020; Ayaz et al. 2019; Ayaz et al. 2020; Ayaz et al. 2018).

### Cell stimulations and treatment with BH

BH was purchased from Saba Pharmaceuticals Ltd. (CAS number: 105979-17-7). J774.2 macrophage cells were plated in 24-well tissue culture plates. In immunostimulation tests, subtoxic concentrations (1, 2, 5, and 10 μg/mL) of BH were added to the appropriate wells in the absence of LPS. For immunomodulatory activity tests, 1 μg/mL of LPS from ENZO Sciences was used per well along with different concentrations of BH: 1, 5, 10, and 20 μg/mL. Wells with only LPS added served as positive control groups, and control groups received no treatment at all. Cell viability was assessed using Trypan blue staining, following the protocol described in our previous studies (Önal et al. 2020; Emen et al. 2022; Önal et al. 2023).

### ELISA measurements

ELISA tests were conducted for cytokine quantification using BD Biosciences kits (CA, USA) for IL12p40 (cat number: 555220), GM-CSF (cat number: 555126), TNF-α (cat number: 555212), and IL-6 (cat number: 555183). A dilution ratio of 1:1000 was applied to both the coating and detection antibodies for each cytokine. The ELISA procedure adhered to the manufacturer's instructions. Coating of 96-well plates was achieved through overnight incubation, followed by three washes with PBS (0.05% Tween). Subsequently, 200 µL of blocking buffer (1% BSA in PBS) was added to each well, and the plates were incubated overnight at 4°C. After three washes, the plates were coated with 100 μl of the detection antibody solution for each cytokine, followed by a 1-hour incubation at room temperature. Following another round of washing, 100 μL of HRP antibody solution was added to each well for a 1-hour incubation at room temperature. After the final wash, 100 μL of TMB substrate (BD OptEIA) was added to each well, and reactions were halted by adding 50 μL of sulfuric acid. Absorbance measurements were recorded at 450 nm (Önal et al. 2023; Önal et al. 2023; Sevin et al. 2023).

### Statistical analysis

Statistical analysis was performed using GraphPad Prism version 5 software. The unpaired two-tailed t-test was employed to assess statistical significance. Each experimental condition was replicated three times (Önal et al. 2023; Önal et al. 2023; Sevin et al. 2023).

## Results

The immunostimulatory activity of BH on macrophages was assessed in the absence of LPS. Cytokine levels were measured without an activator to investigate the potential stimulatory role of BH. Additionally, the immunomodulatory activity of BH was examined by incubating LPS-activated macrophages with varying concentrations of BH. The modulatory role of BH on these cells was determined based on changes in pro-inflammatory cytokine production levels by the activated macrophages.

**Fig. 1:**
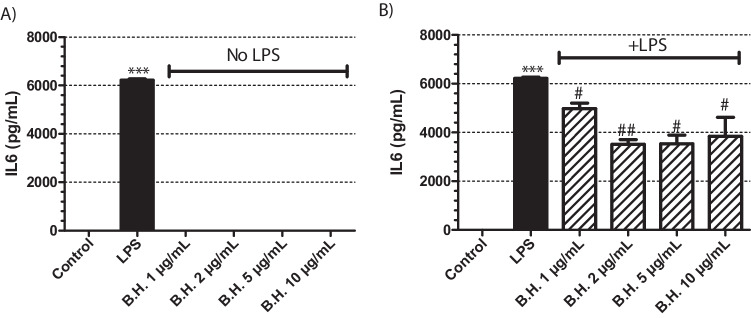
Effect of Benipidine on IL6 release: The cells were treated with BH at concentrations of 1, 2, 5, and 10 μg/mL for 24 hours (**A**) in the absence of LPS and (**B**) presence of 1 μg/mL LPS. Positive control group had only 1 μg/mL LPS treatment. The sample size for each set was 3 (N = 3). p values are represented for BH treated groups comapared to the control group in (**A**) when the samples were analyzed in the absence of LPS, whereas in samples with LPS stimulation (**B**), the p values were are represented compared to the only LPS treated positive control samples. p value for only LPS treated sample is represented in comparison to non-treated control group in order to show that the positive control worked (Significance level: *p<0.01, ** p<0.005, *** p<0.001 vs control; #p<0.01, ##p<0.005, ###p<0.001 vs LPS. N=3, All data are expressed as means ± S.E.M. of 3 independent experiments)

Macrophages were treated with a concentration range of BH for 24 hours. BH was not able to stimulate the macrophages to produce IL-6 while positive control only LPS treated group had substantial amounts of IL-6 (Fig 1A). Therefore, it lacked immunostimulatory activity.

In order to test the immunomodulatory activity of BH, it was given together with LPS to the macrophages. After 24 hours of incubation period, the supernatants were analyzed by ELISA for IL-6 levels. Compared to the positive control only LPS treated groups, there was a substantial and significant decrease in the IL-6 production of the macrophages in the presence of BH (Fig. 1B). Therefore, BH had anti-inflammatory immunomodulatory activity.

**Fig. 2:**
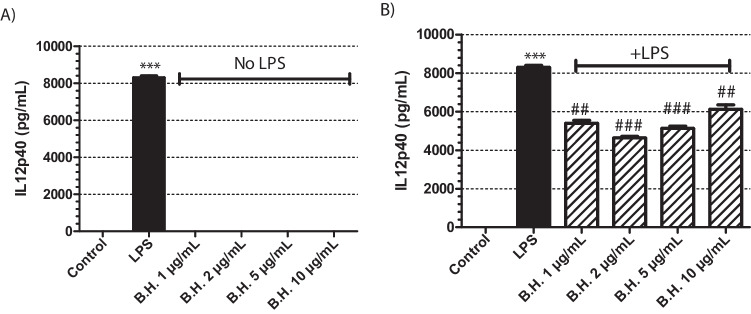
Effect of Benipidine on IL12p40 release: The cells were treated with BH at concentrations of 1, 2, 5, and 10 μg/mL for 24 hours (**A**) in the absence of LPS and (**B**) presence of 1 μg/mL LPS. Positive control group had only 1 μg/mL LPS treatment. The sample size for each set was 3 (N = 3). p values are represented for BH treated groups comapared to the control group in (**A**) when the samples were analyzed in the absence of LPS, whereas in samples with LPS stimulation (**B**), the p values were are represented compared to the only LPS treated positive control samples. p value for only LPS treated sample is represented in comparison to non-treated control group in order to show that the positive control worked (Significance level: *p<0.01, ** p<0.005, *** p<0.001 vs control; #p<0.01, ##p<0.005, ###p<0.001 vs LPS. N=3, All data are expressed as means ± S.E.M. of 3 independent experiments)

IL12p40 ELISA was done to the supernatants of the samples that were treated with BH in the absence of LPS (Fig. 2A). BH was not able to stimulate the cells to produce IL12p40 (Fig 2A). Whereas when the cells were activated with LPS and treated with BH, BH led to a significant decrease in IL12p40 production compared to the only LPS treated positive control groups (Fig 2B).

**Fig. 3:**
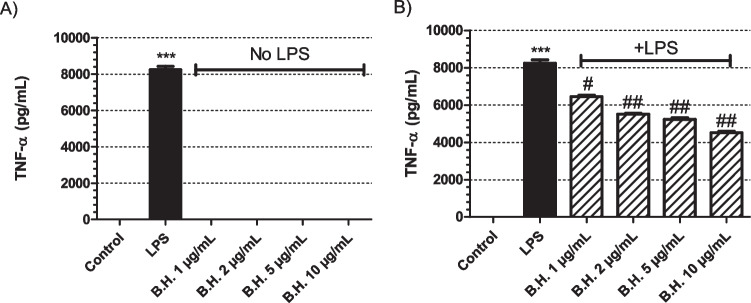
Effect of Benipidine on TNF-α release: The cells were treated with BH at concentrations of 1, 2, 5, and 10 μg/mL for 24 hours (**A**) in the absence of LPS and (**B**) presence of 1 μg/mL LPS. Positive control group had only 1 μg/mL LPS treatment. The sample size for each set was 3 (N = 3). p values are represented for BH treated groups comapared to the control group in (**A**) when the samples were analyzed in the absence of LPS, whereas in samples with LPS stimulation (**B**), the p values were are represented compared to the only LPS treated positive control samples. p value for only LPS treated sample is represented in comparison to non-treated control group in order to show that the positive control worked (Significance level: *p<0.01, ** p<0.005, *** p<0.001 vs control; #p<0.01, ##p<0.005, ###p<0.001 vs LPS. N=3, All data are expressed as means ± S.E.M. of 3 independent experiments)

TNFα ELISA was done for the supernatants of the BH treated macrophages in the absence of LPS. BH was not able to stimulate the production of this cytokine by itself (Fig 3A). Whereas, in the presence of LPS there was a substantial decrease in the production of TNFα cytokine when the cells were treated with different concentrations of BH compared to the only LPS treated groups and this difference was significant (Fig 3B).

**Fig. 4:**
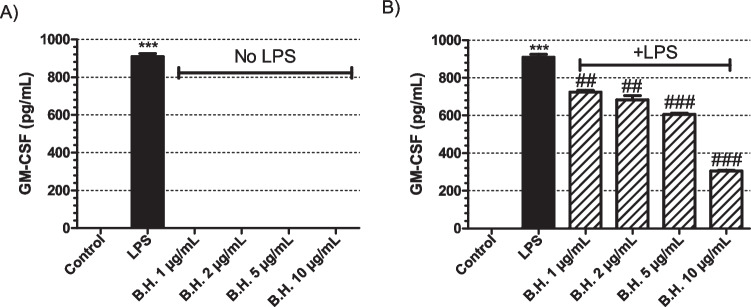
Effect of Benipidine on GM-CSF release: The cells were treated with BH at concentrations of 1, 2, 5, and 10 μg/mL for 24 hours (**A**) in the absence of LPS and (**B**) presence of 1 μg/mL LPS. Positive control group had only 1 μg/mL LPS treatment. The sample size for each set was 3 (N = 3). p values are represented for BH treated groups comapared to the control group in (**A**) when the samples were analyzed in the absence of LPS, whereas in samples with LPS stimulation (**B**), the p values were are represented compared to the only LPS treated positive control samples. p value for only LPS treated sample is represented in comparison to non-treated control group in order to show that the positive control worked (Significance level: *p<0.01, ** p<0.005, *** p<0.001 vs control; #p<0.01, ##p<0.005, ###p<0.001 vs LPS. N=3, All data are expressed as means ± S.E.M. of 3 independent experiments)

GMCSF production was measured in the absence of LPS when the macrophages were treated with different concentrations of BH (Fig 4A). BH was not able to stimulate the production of GMCSF by itself (Fig 4A). When the cells were activated with LPS, BH was significantly suppressing the production of GMCSF (Fig 4B).

Overall, these results suggest that BH has anti-inflammatory immunomodulatory activity while lacking any immunostimulatory activity.

## Dıscussıon

As a member of the calcium channel blockers (CCBs) class, BH is commonly prescribed as a first-line medication for the management of hypertension (McDonough et al. 2020). However, limited research has been conducted on the impact of BH on the immune system and macrophage cells. Understanding this information is crucial for clinical decision-making when prescribing the drug to patients. In cases where a patient has hypertension accompanied by inflammation, the effect of BH on inflammation becomes pivotal in determining the appropriate treatment strategy. Our results suggest that BH itself, without the need for additional anti-inflammatory agents, may be effective since it exhibits anti-inflammatory activity on mammalian macrophages.

Previous studies have provided insights into the effects of BH on the immune system. Li et al. delved into the inhibitory mechanism of BH against Severe Fever with Thrombocytopenia Syndrome Virus (SFTSV) infection, exploring its potential impact on viral internalization and binding. The findings indicated that BH effectively inhibited the internalization and genomic replication of SFTSV. However, it did not demonstrate any impact on virus binding, fusion, or budding. It is essential to note that additional research is required to obtain definitive evidence. During their investigation, Li et al. also observed that no BH-resistant virus was detected, even after subjecting the virus to more than 20 passages in serial culturing while being exposed to BH (Li et al. 2019).

In a study conducted by Çakır et al., the neuroprotective properties of BH against cerebral ischemia/reperfusion (I/R) injury in rats were examined. The investigation revealed an increase in COX-2 levels in the cerebral cortex tissues following ischemia/reperfusion (I/R) injury. However, after the administration of BH, COX-2 levels returned to normal, indicating its inhibitory effects. This phenomenon may be attributed to the prevention of calcium ion influx into cells during ischemia. Moreover, BH is believed to possess distinct protective properties against cerebral ischemia/reperfusion (I/R) injury, potentially due to its ability to inhibit the excessive production of inflammatory cytokines and enhance antioxidant capacity in brain tissue (Çakır et al. 2021). COX-2 is recognized as a crucial protein in the generation of inflammatory reactions (Zarghi and Arfaei 2011).

In a study conducted by Wang et al., the effectiveness of BH in preventing infections caused by hantaviruses was investigated. The study observed that BH demonstrated preventive properties against hantavirus infections. To assess the inhibitory effects of BH on various hantaviruses, Wang et al. isolated VSV pseudoviruses coated with pathogenic hantaviral glycoproteins, including SNV, SEOV, ANDV, DOBV, and PUUV. The results revealed a significant reduction in the infection rates of these pseudoviruses, suggesting that BH possesses a broad-spectrum anti-hantavirus activity. The study concludes that BH holds promise as a potential therapeutic approach for addressing hantavirus infections (Wang et al. 2022).

In light of these studies, our aim was to examine the impact of BH on macrophage cells in an in vitro inflammation model. The study involved evaluating the levels of IL-12p40, TNF-α, GM-CSF, and IL-6 cytokines in J774.2 macrophage cells after a 24-hour incubation with BH, both in the presence and absence of LPS. In the absence of LPS, we investigated whether BH alone could stimulate macrophages to produce TNFα, IL-6, GM-CSF, and IL-12p40. Our results suggest that BH lacked immunostimulatory activity, as it did not activate macrophages for the production of these pro-inflammatory cytokines (Figs. 1A, 4A). In a clinical setting, this implies that BH treatment would not exacerbate inflammation in the body.

BH was administered to the macrophages in the presence of LPS, mimicking an ongoing inflammation in the body through an in vitro setting. A substantial and significant decrease in the production of pro-inflammatory cytokines—TNFα, IL-6, GM-CSF, and IL12p40—was observed (Figs. 1B, 4B). This implies that in a clinical setting, hypertension patients with inflammatory reactions can be prescribed BH alone, eliminating the need for additional anti-inflammatory drugs (Patrick et al. 2021; De Miguel et al. 2015; Xiao and Harrison 2020). Such an approach not only simplifies treatment but also helps prevent drug overdosage and possible side effects (Martinez et al. 2023; Sofy et al. 2020; Matsunaka et al. 2022; Umemoto et al. 2017; Xue et al. 2017).

## Conclusıon

Understanding the effects of drugs on immune system cells is crucial, especially considering that many diseases are associated with inflammation. If a drug molecule exacerbates inflammatory reactions, it can complicate the patient's situation while treating other symptoms (Patrick et al. 2021; De Miguel et al. 2015; Xiao and Harrison 2020). Conversely, a drug molecule with anti-inflammatory activity, alongside other specific functions, can eliminate the need for additional anti-inflammatory drugs when inflammation accompanies other disease symptoms. Our study focused on a commonly prescribed hypertension drug, BH, which exhibited anti-inflammatory activities without any immunostimulatory effects. This implies that patients suffering from hypertension along with inflammation may not require extra anti-inflammatory drug usage. Such an approach can help prevent possible side effects due to the overuse of drugs. Further studies with versatile immune system cell types are needed to fully decipher the effects of this drug molecule on the immune system and its potential impact on patient well-being.

## Data Availability

The data is available from the corresponding author upon reasonable request.
